# Supplementary material for: Multi-state data storage in a two-dimensional stripy antiferromagnet implemented by magnetoelectric effect
Source: Nat Commun. 2023 Jun 3;14:3221. doi: 10.1038/s41467-023-39004-4 (PMC10239514; doi:10.1038/s41467-023-39004-4)
Supplement: Supplementary file 1 — Supplementary Information [file 41467_2023_39004_MOESM1_ESM.pdf]

Supplementary Information for
Multi-state Data Storage in a Two-dimensional Stripy Antiferromagnet Implemented by Magnetoelectric Effect

Pingfan Gu[1,2], Cong Wang[3], Dan Su[4], Zehao Dong[1], Qiuyuan Wang[1], Zheng Han[5,6], Kenji Watanabe[7], Takashi Taniguchi[8], Wei Ji[3,⋆], Young Sun[9,⋆] and Yu Ye[1,2,10,⋆]

[1] *State Key Laboratory for Mesoscopic Physics and Frontiers Science Center for Nano-optoelectronics, School of Physics, Peking University, Beijing, 100871, China*
[2] *Collaborative Innovation Center of Quantum Matter, Beijing 100871, China*
[3] *Department of Physics and Beijing Key Laboratory of Optoelectronic Functional Materials and Micro-Nano Devices, Renmin University of China, Beijing 100872, China*
[4] *Beijing National Laboratory for Condensed Matter Physics, Institute of Physics, Beijing 100190, China*
[5] *State Key Laboratory of Quantum Optics and Quantum Optics Devices, Institute of Optoelectronics, Shanxi University, Taiyuan 030006, China*
[6] *Collaborative Innovation Center of Extreme Optics, Shanxi University, Taiyuan 030006, China*
[7] *Research Center for Functional Materials, National Institute for Materials Science, Tsukuba 305-0044, Japan*
[8] *International Center for Materials Nanoarchitectonics, National Institute for Materials Science, Tsukuba 305-0044, Japan*
[9] *Center of Quantum Materials and Devices, and Department of Applied Physics, Chongqing University, Chongqing 400044, China*
[10] *Yangtze Delta Institute of Optoelectronics, Peking University, Nantong 226010 Jiangsu, China*
⋆ *Email: wji@ruc.edu.cn, youngsun@cqu.edu.cn, ye_yu@pku.edu.cn*

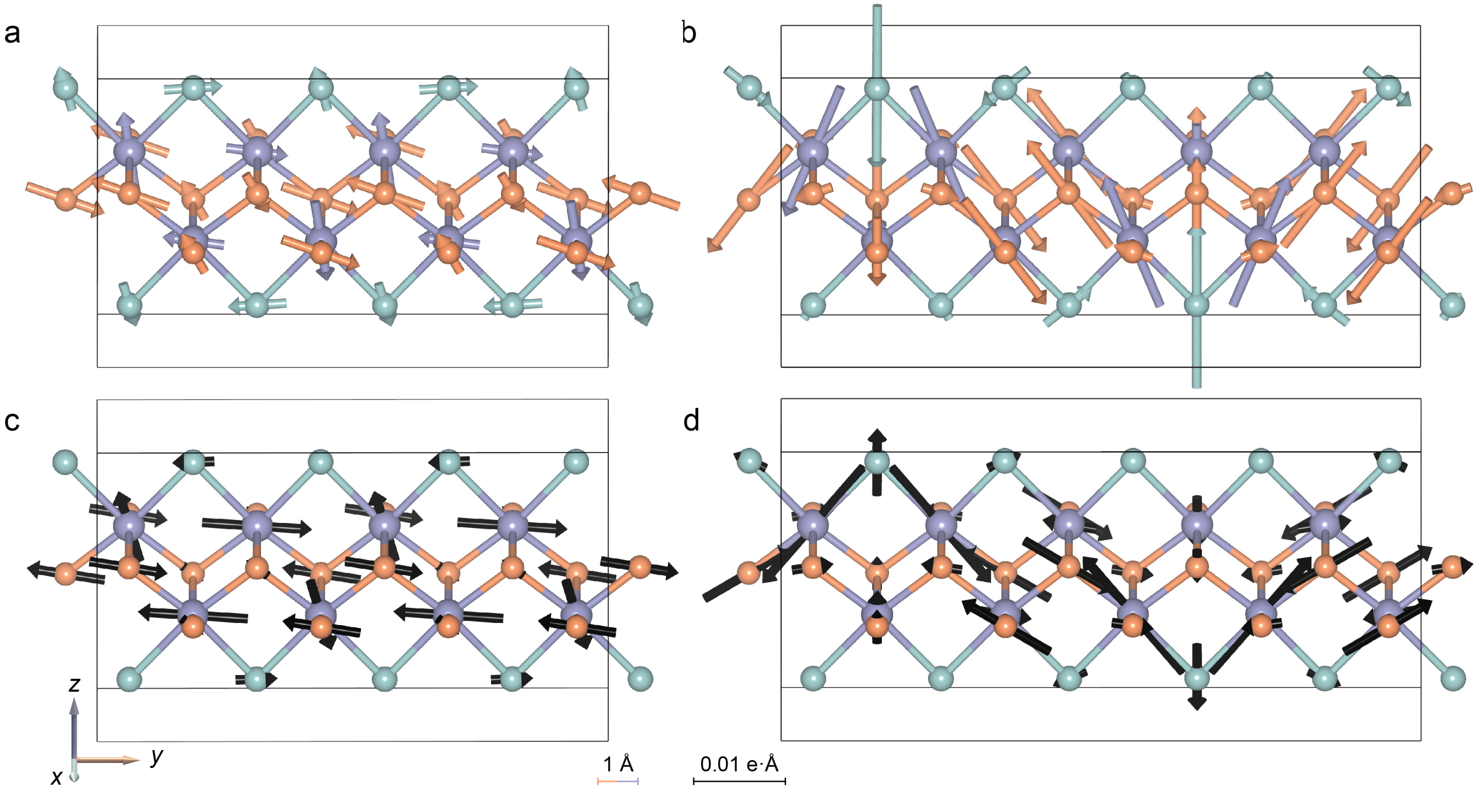

Fig. S1 Atomic displacements and dipoles of CrOCl. (a, b), The atomic distortion configurations of CrOCl in the ↑↑↓↓ (a) and the ↑↑↑↓↓ (b) phases. The vectors on each atom represent the atomic displacements which are magnified 100 times. **(c, d),** The dipole configurations of CrOCl in the ↑↑↓↓ (c) and the ↑↑↑↓↓ (d) phases. Black arrows depict theoretical predictions of the electric polarization of each atom by the Born effective charge method. The scale bars of the Cartesian coordinates and the electric dipoles are plotted at the bottom.

Table S1 Calculated space group, lattice parameters and net electric dipole under the external electric field in the ↑↑↓↓ and ↑↑↑↓↓ phases of CrOCl. The electric dipole was obtained under an external field of 0.07 V/Å along the $+z$-direction in a single unit cell.

	↑↑↓↓	↑↑↑↓↓
Structure	Monoclinic $P2_1/m$	Orthorhombic $Pmmn$
Unit cell	(a, 4b c)	(a, 5b, c)
a (Å)	3.89413	3.89458
b (Å)	3.19511	3.19648
c (Å)	7.67976	7.67714
α (°)	90.0627	90.0000
V (Å^3)	382.211636	477.861592
P (e · Å)	0.00278	0.00043

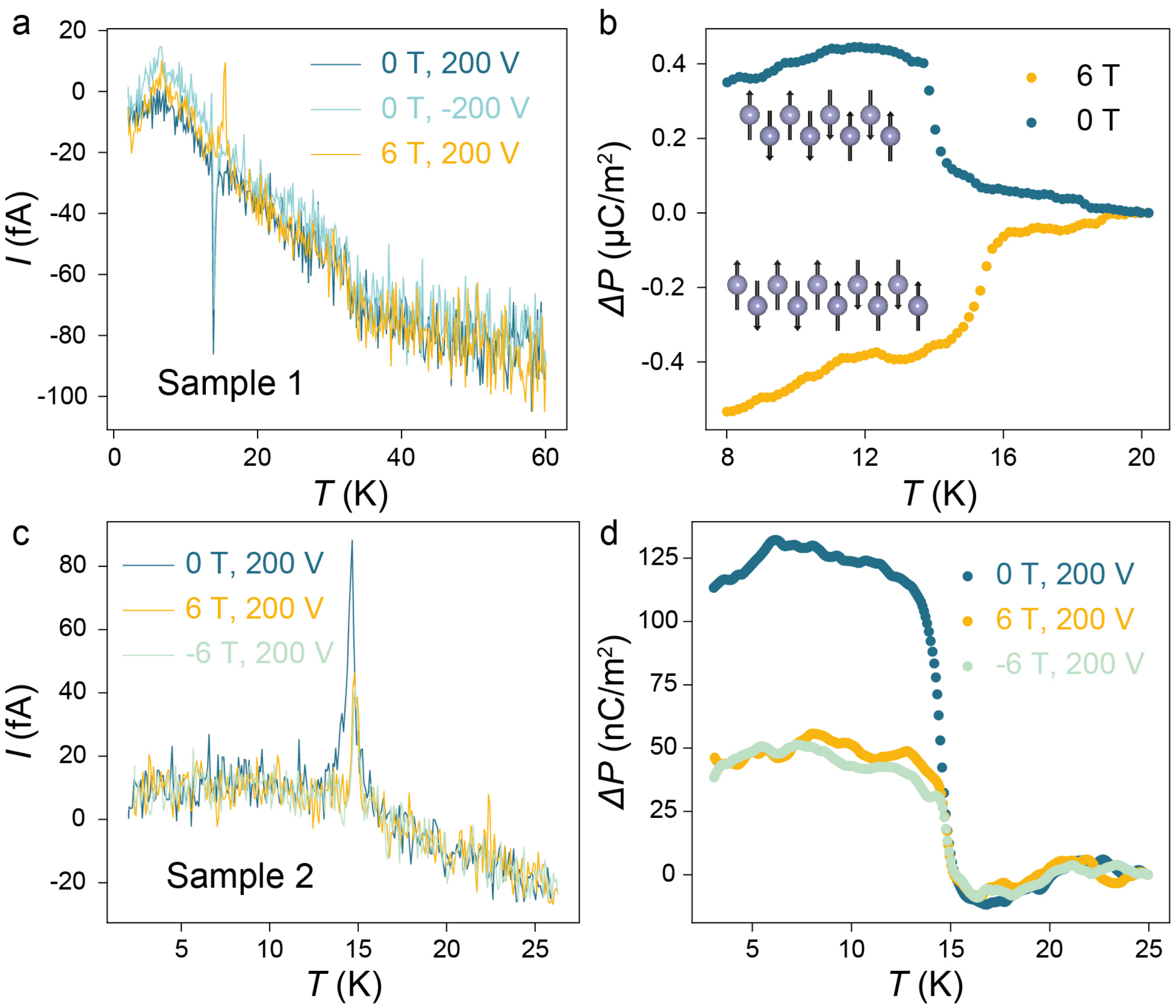

Fig. S2 Temperature-dependent polarization of CrOCl crystals. **(a)** and **(c)** show the pyroelectric current *versus* temperature of sample 1 and sample 2 with different magnetic fields or poling electric fields. **(b)** and **(d)** show the corresponding electric polarization ΔP integrated from **(a)** and **(c)**. The critical temperatures agree well with the magnetic phase diagram of CrOCl[1].

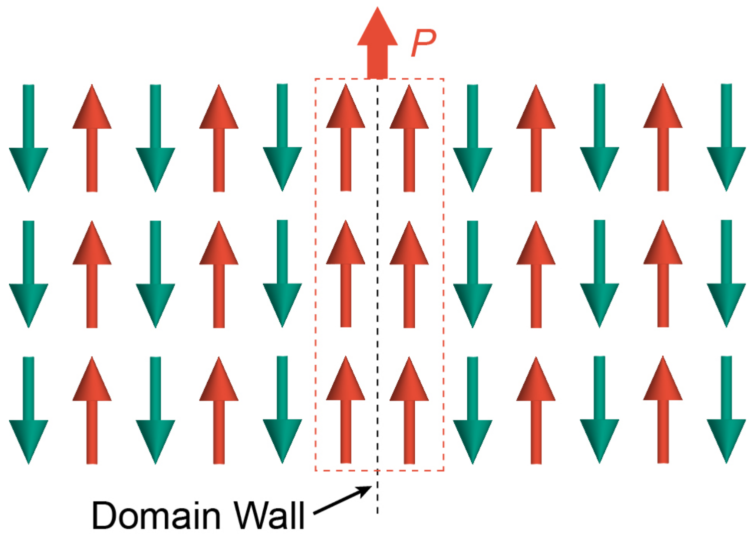

Fig. S3 Illustration of the domain wall induced local net polarization in antiferroelectric materials.

To explain the origin of the measured net polarization, we would like to first clarify the details of the pyroelectric measurements. We painted silver epoxy on both sides of the bulk sample, with an area of $\sim$ 3 mm^2, to serve as electrodes. We first lowered the ambient temperature to 2 K with a poling field of 200 V, then removed the external field and measured the pyroelectric current versus time as the

temperature increases. As a result, the pyroelectric current detects the spontaneous polarization of the crystal covered by the electrodes. The change of net polarization P over time causes the motion of the interfacial charge, so the detectable current can be expressed by:

$$I = \frac{dQ}{dt} = \frac{d(P \cdot A)}{dt} = A \cdot \frac{dP}{dt} \tag{S1}$$

where A is the area of the junction. We here present the data from two different samples. We note that in both samples, the $\uparrow\uparrow\downarrow\downarrow$ and the $\uparrow\uparrow\uparrow\downarrow\downarrow$ states exhibit detectable but very tiny pyroelectric current ($\sim 10^{-14}$ A) at the critical temperature. The measured polarizations were identical under the external field of +6 T and -6 T (Fig. S2d), ruling out the possibility of artifacts in measurements, as the electric polarization should remain unchanged under the time-reversal operation. However, as shown in Fig. S2a, the current does not change sign when the poling voltage becomes negative. Obviously, this behavior cannot be explained by spontaneous polarization, but is fairly common in structural phase transitions with a large number of domains. As illustrated in Fig. S3, when a domain wall appears inside the antiferroelectric order, the inversion symmetry is locally broken, giving rise to finite electric polarization. In practice, multiple grain boundaries should exist in our CVT-grown sample within a contact area of ~ 3 mm^2. The measured pyroelectric current, consequently, should be the integration of all the tiny net polarizations induced at the grain boundaries. Considering the randomness of domain structure and the evolution of the domain walls over time, the value of the integration result should be random. Since both the $\uparrow\uparrow\downarrow\downarrow$ and the $\uparrow\uparrow\uparrow\downarrow\downarrow$ phases undergo structural phase transitions and exhibit periodic electric dipole structures (Fig. S1), they are both expected to exhibit small pyroelectric current with random values. This randomness is reflected in the difference between sample 1 and sample 2, where not only the polarization values are different, but the polarization directions of the $\uparrow\uparrow\uparrow\downarrow\downarrow$ state are also opposite to each other.

Although the exact value of pyroelectric current is unimportant, its presence is rather convictive. We therefore present the pyroelectric measurement results here as supporting evidence that no spontaneous polarization but antiferroelectric order appears after the magnetic phase transitions, in contrast to the dielectric measurements which is sensitive to the dynamic properties of the electric dipoles *via* A.C. current. Pyroelectric measurements demonstrate the appearance of the antiferroelectric order in the $\uparrow\uparrow\downarrow\downarrow$ and the $\uparrow\uparrow\uparrow\downarrow\downarrow$ states, while dielectric measurements confirm the adjustability of the antiferroelectric dipoles, both of which are consistent with our DFT calculations.

Compared to the pyroelectric measurements, the tunneling resistance of nanodevices is influenced by the antiferroelectric order in a completely different way. On the one hand, the area of the tunneling junction is $\sim$ μm^2, which is smaller than the typical domain size of 2D magnetic materials[2], so we tend to believe that the tunneling junction is initially composed of a single domain with homogeneous antiferromagnetic and antiferroelectric structure. As a result, the tiny net polarization caused by the domain walls should not affect the tunneling resistance. On the other hand, the pyroelectric measurements are performed at zero bias voltage, while the tunneling currents are measured at finite bias voltages, which means a large electric field. Therefore, the electric polarization that plays a role in electron tunneling should mainly result from the tilting of the antiferroelectric dipoles under large electric fields.

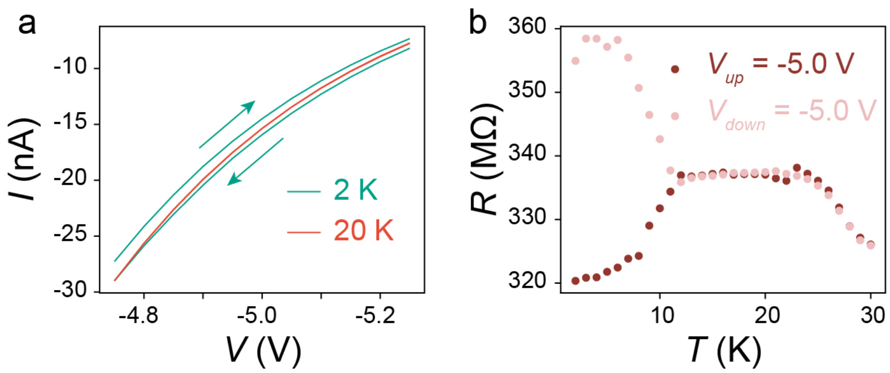

Fig. S4 Hysteresis behavior at negative voltages. (a), $I - V$ curves of the device 1 at 20 K (above the Néel temperature) and 2 K (below the Néel temperature). **(b),** Resistance *versus* temperature of the CrOCl tunneling device at -5 V in different sweeping processes. Data were collected from the same device as Fig. 2 in the main text.

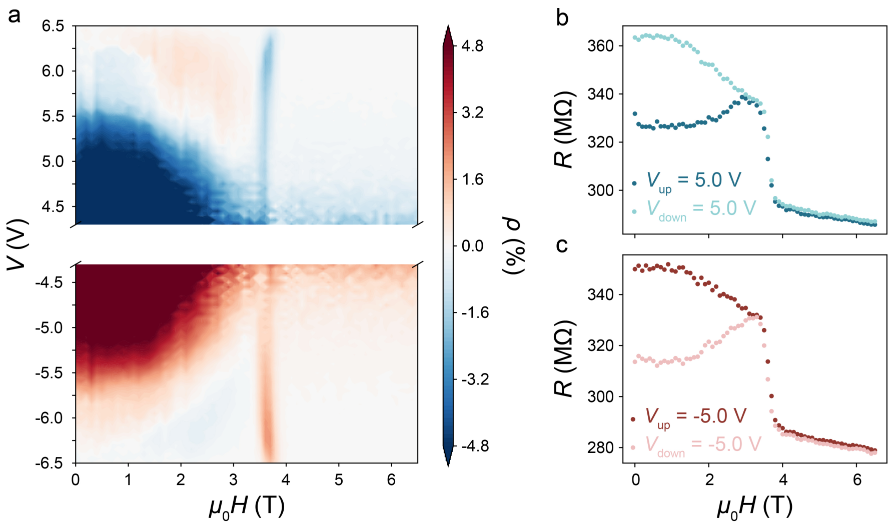

Fig. S5 $I - V$ **hysteresis of device 1 at 2 K under out-of-plane external field. (a),** 2D map of current polarization ρ as a function of out-of-plane magnetic field and bias voltage. **(b,c),** Tunneling resistance *versus* out-of-plane magnetic field at 5.0 V (b) and -5.0 V (c) in different sweeping processes.

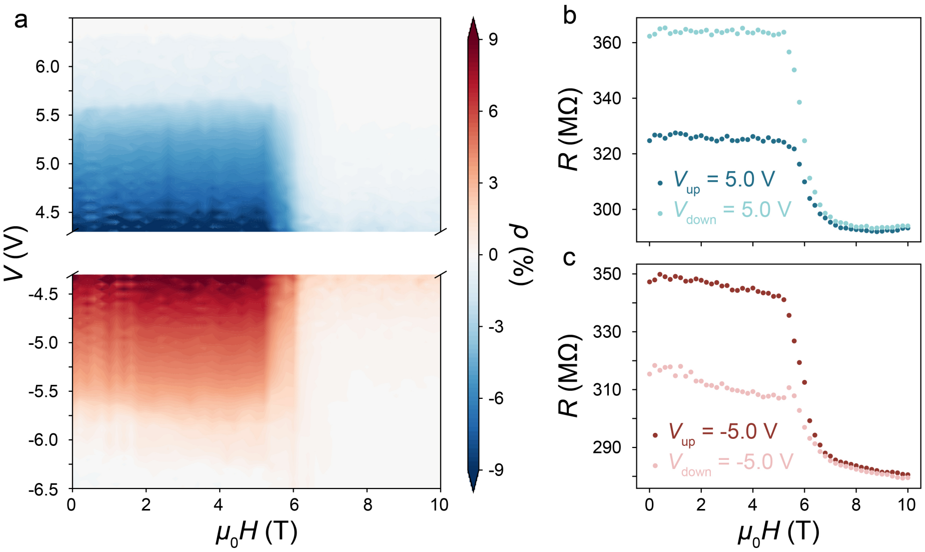

Fig. S6 $I - V$ **hysteresis of device 1 at 2 K under in-plane external field. (a),** 2D map of current polarization ρ as a function of in-plane magnetic field and bias voltage. **(b,c),** Tunneling resistance *versus* in-plane magnetic field at 5.0 V (b) and -5.0 V (c) in different sweeping processes.

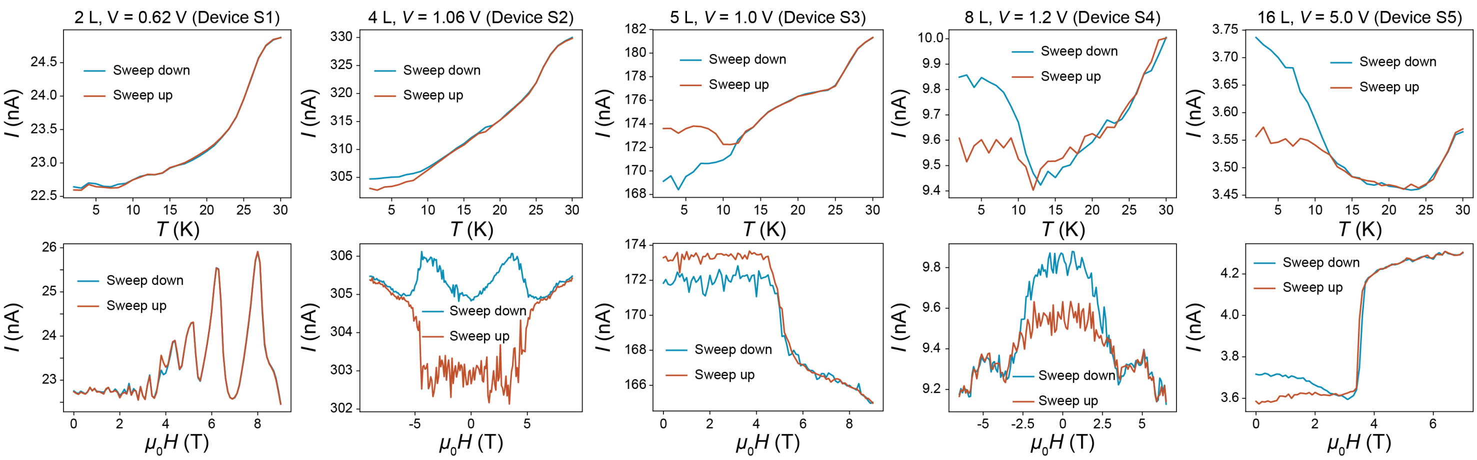

Fig. S7 $I-V$ **hysteresis observed in devices with different thicknesses.** Although the specific behaviors vary in different devices, the critical temperature precisely accords with the Néel temperature in all devices and the hysteresis disappears after the transition to the ↑↑↑↓↓ state under the external magnetic field.

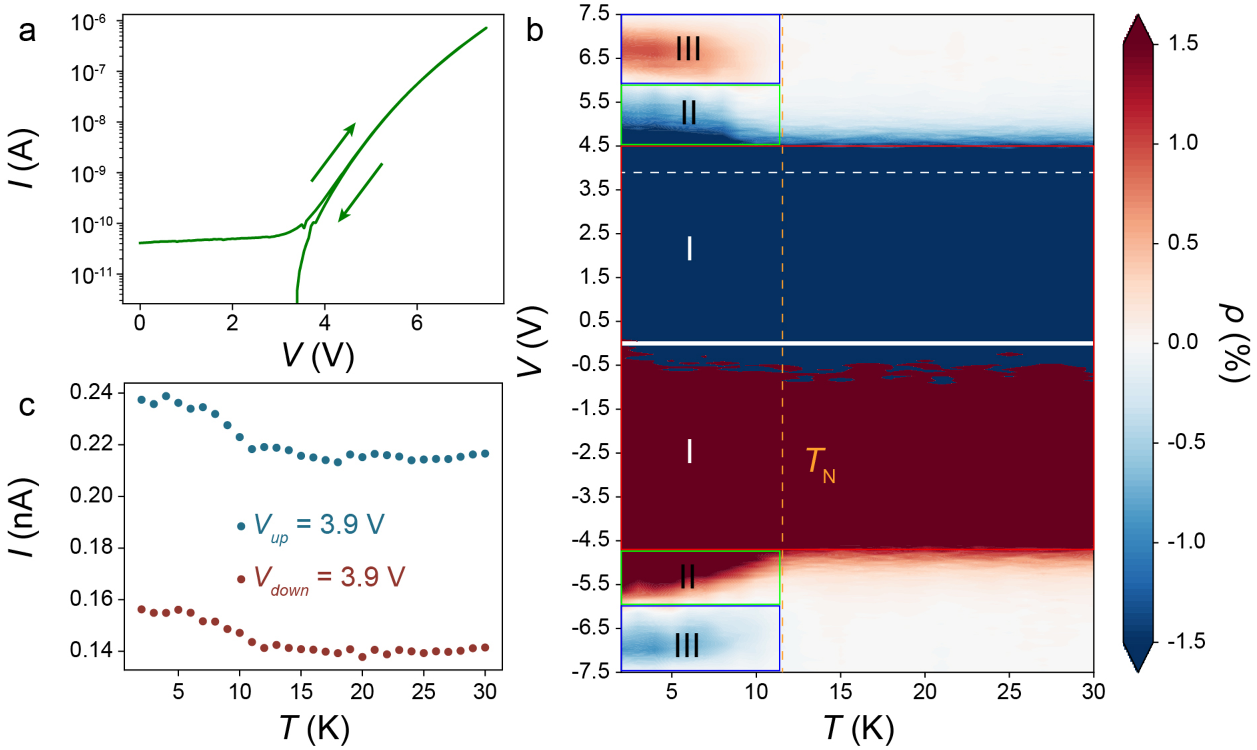

Fig. S8 $I-V$ **hysteresis of device 3 (**$\sim$ 11.3 nm **CrOCl). (a),** $I-V$ curve of the device in semi-logarithmic coordinates. **(b),** 2D map of current polarization $\rho = (R_{\text{up}} - R_{\text{down}})/(R_{\text{up}} + R_{\text{down}})$ as a function of temperature and bias voltage. The vertical orange dashed line marks the Néel temperature of the exfoliated CrOCl. The horizontal white dashed line marks the bias voltage of 3.9 V. The $I-V$ hysteresis is divided into three regions, namely I, II and III, enclosed by red, green and blue boxes, respectively. **(c),** Device tunneling current *versus* temperature at 3.9 V in different sweeping processes.

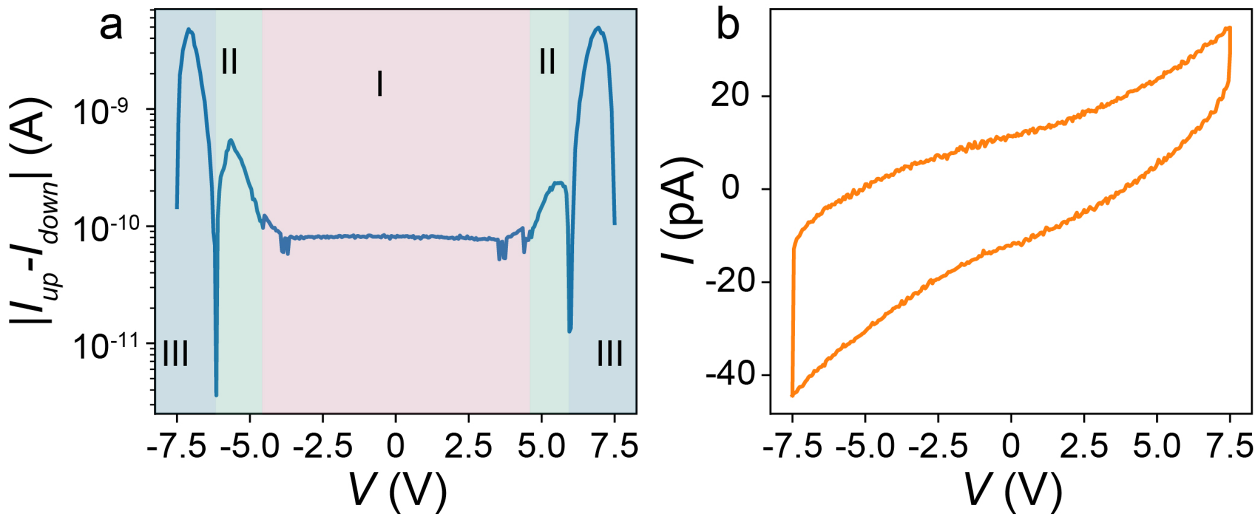

Fig. S9 **(a),** Difference in tunneling current $|I_{\text{up}} - I_{\text{down}}|$ *versus* bias voltage at 2 K extracted from device 3. The curve is divided into three regions by three partitions of different background colors that represent different physical mechanisms of the hysteresis, corresponding to the three regions labelled in Fig. S8b. **(b),** $I - V$ curve measurement of the empty system under the same measurement conditions as the data presented in the manuscript. We note that the tiny hysteresis has a negligible effect on the intrinsic hysteresis with the order of nA.

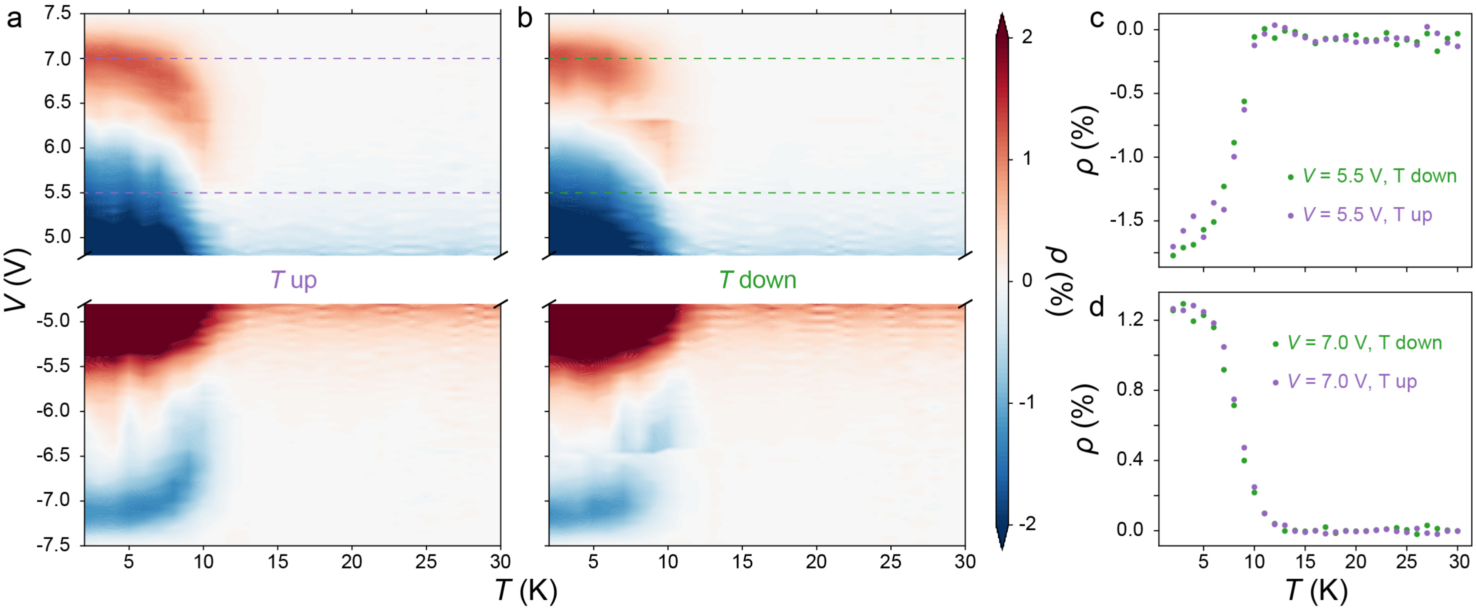

Fig. S10 $I - V$ **hysteresis hysteresis in** T **up and** T **down processes of device 3.** **(a),** 2D map of current polarization ρ in the warm-up process. **(b),** 2D map of ρ in the cooling process. The dashed lines mark the bias voltages of 5.5 V and 7.0 V. **(c, d),** ρ *versus* temperature in the T up and T down processes at voltages of 5.5 V (c) and 7.0 V (d). The tunneling current and polarization show no obvious changes.

Here, we exclude extrinsic origins that might cause the $I - V$ hysteresis in the CrOCl tunneling devices. To show the difference between the hysteresis caused by intrinsic and extrinsic factors, we replotted the 2D map of the current polarization in Fig. S8 over the entire voltage range. Data were collected from device 3. The tunneling current at low voltages exhibits a large hysteresis loop that persists above the Néel temperature and is almost temperature-independent (Fig. S8b), which is not related to the phase transition in CrOCl and we attribute to extrinsic contributions. We take the $I - T$ curves of different sweeping processes at 3.9 V as an example (Fig. S8c). The tunneling current exhibits an almost constant difference ($\sim$ 0.08 nA) between the sweep-up and sweep-down processes as the temperature varies. There are two reasons that possibly explain this hysteresis loop at low voltages: the charging current of stray capacitances in the measurement circuit (Fig. S9b) and the charge traps. Both are unrelated to the antiferroelectric order of CrOCl and therefore meaningless to our study. It should be noted that these effects persist in all voltage ranges, but only provide a hysteresis of about about a few 10s pA and are therefore negligible at higher voltages.

To protect our device from large local thermal effect, we limited the electric power to below 10 μW, so the sweeping voltage range is from -7.5 V to 7.5 V. The hysteresis loop should enclose at the sweeping terminals, which means that the current polarization must be zero around ±7.5 V. Consequently, the most salient features can be found in the voltage range between 4.5 V and 7.5 V, where the current is measurable and is believed to be dominated by the quantum tunneling mechanism, as shown in Fig. S9a. Depending on the thickness of the tunneling

barrier and the area of the tunneling junction, the crucial voltage range will vary from device to device. Based on the above discussions, the whole curve is divided into three regions: I, II and III, labelled correspondingly in Fig. S8b and Fig. S9a. Region I represents the clockwise hysteresis resulted from extrinsic factors. Region II and III label the clockwise and counterclockwise hysteresis resulted from the intrinsic tilting of the antiferroelectric dipoles, which contain the most physical information and are the main focus of our study. The zoom-in figure and the relaxation process of Region II and Region III are presented in Fig. S11 and Fig. S12, respectively. The possible explanations will be discussed by the following simulation results.

In total, we measured more than twenty devices, and the $I-V$ hysteresis appears in devices of all thicknesses and exhibited similar behaviors. In Fig. S7, we present five other devices with different thicknesses in addition to the devices in the main text. The most important feature that distinguishes it as an intrinsic characteristic is that it accompanies the magnetic transitions. The hysteresis only appears below the Néel temperature and disappears under the external magnetic field after the transition to the ↑↑↑↓↓ state (Fig. S5 and S6), consistent with our conjecture that there are no tiltable electric dipole in the ↑↑↑↓↓ state (indicated by the dielectric measurements and DFT calculations). We also ruled out any extrinsic possibilities such as electromigration, oxygen vacancy redistribution or charge trapping as there is no reason that they could reproducibly appear in all devices and would not degenerate after multiple temperature or magnetic rises and falls (Fig. S10). Furthermore, the ↑↑↓↓ stripy antiferromagnetic phase is an ordered state and minimized in free energy proved by DFT calculations[1,3], so it cannot be explained why the charge traps play a role in the ↑↑↓↓ phase but not in the ↑↑↑↓↓ state and the paramagnetic state. In conclusion, we find the tilting of electric dipoles to be the only possible and the most plausible physical mechanism to explain the $I-V$ hysteresis.

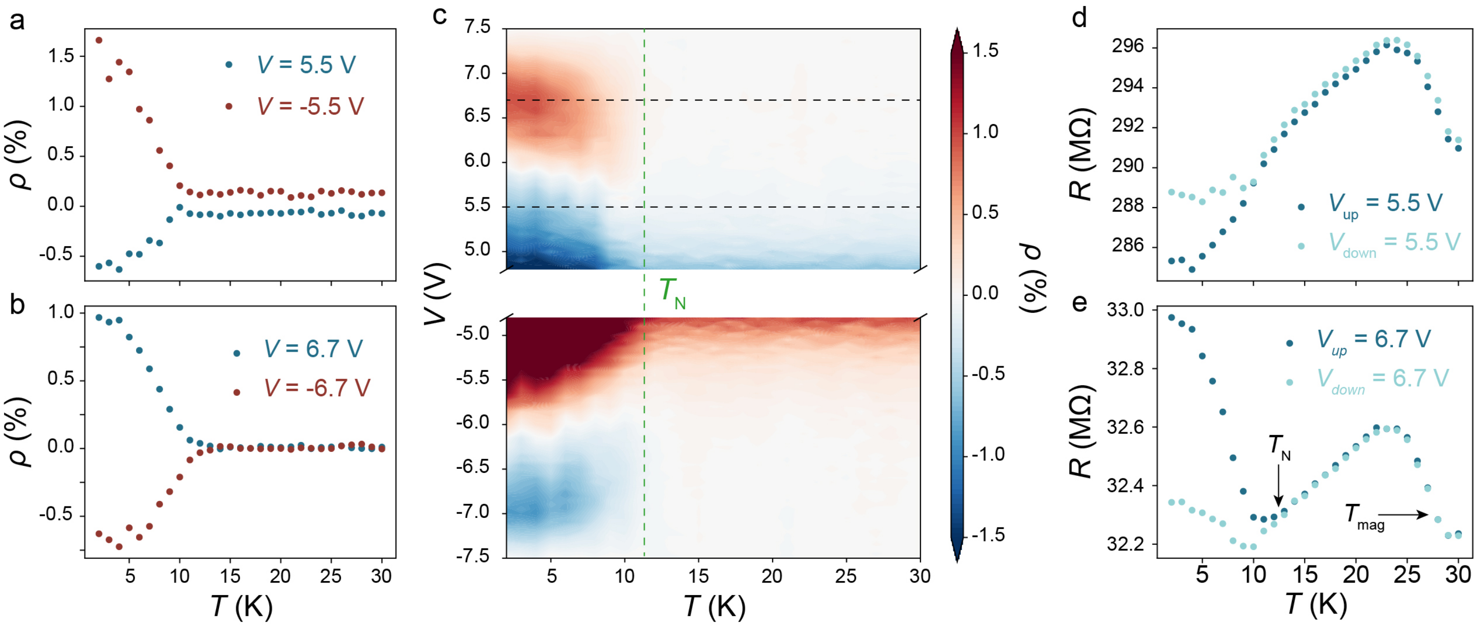

Fig. S11 $I - V$ **hysteresis of device 3 below Néel temperature.** **(a,b),** Current polarization $\rho = (R_{up} - R_{down})/(R_{up} + R_{down})$ *versus* temperature at ±5.5 V (a) and ±6.7 V (b). **(c),** 2D map of current polarization ρ as a function of temperature and bias voltage. The vertical green dashed line marks the Néel temperature of the exfoliated CrOCl. The horizontal black dashed lines mark the bias voltages of 5.5 V and 6.7 V. **(d,e),** Resistance *versus* temperature of the CrOCl tunneling device at 5.5 V (d) and 6.7 V (e) in different sweeping processes.

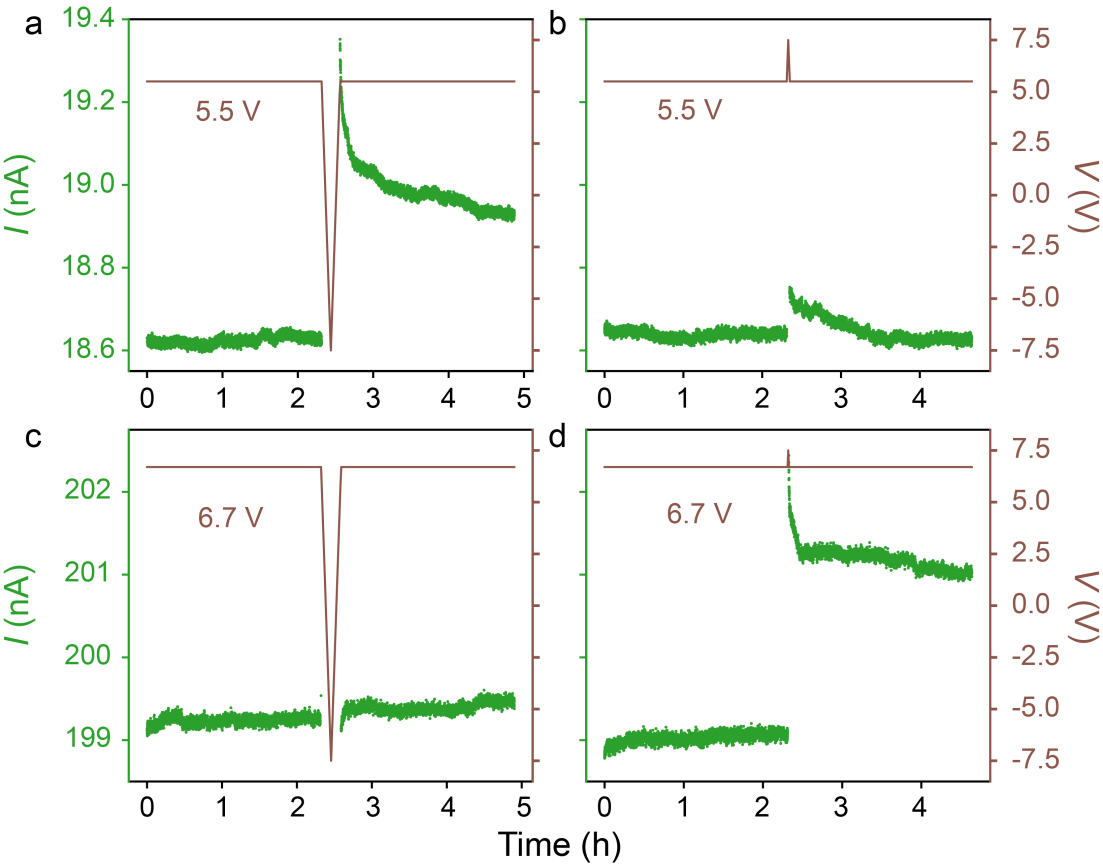

Fig. S12 Tunneling current relaxation process at different voltages of device 3. **(a,b),** Time-dependent relaxation curves at 5.5 V in the sweep-up (a) and sweep-down (b) processes. **(c,d),** Time-dependent relaxation curves at 6.7 V in the sweep-up (c) and sweep-down (d) processes..

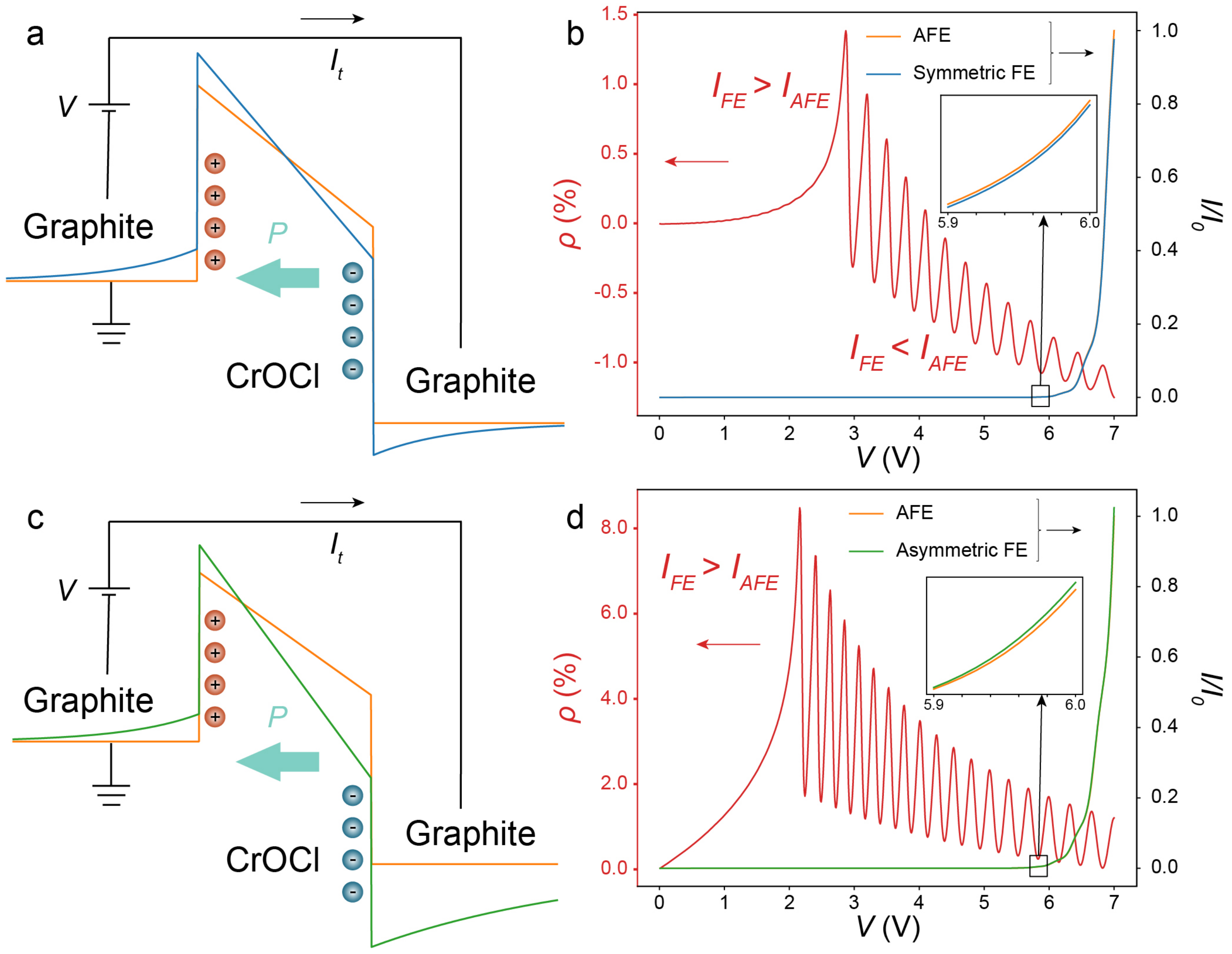

Fig. S13 The theoretical model of the electron tunneling process. (a,c), Energy profile of the unpolarized AFE state and the polarized FE state with symmetric graphite electrodes (a) and asymmetric graphite electrodes (c). **(b, d),** Calculated tunneling current of the AFE state and the FE state and the current polarization ρ with symmetric graphite electrodes (b) and asymmetric graphite electrodes (d). The tunneling current values are normalized dividing by the maximum current of the antiferroelectric state. The insets are the zoomed-in views of the tunneling current at 5.9 V $\leq V \leq$ 6.0 V.

We now propose a theoretical model to simulate the $I - V$ curves of CrOCl tunneling devices. The electric polarization influences the electron tunneling process mainly through three effects: the electrostatic effect, the interface effect and the strain effect, among which the electrostatic effect is considered to be the most decisive[4–7]. As shown in Fig. S13a, since the graphite stripes we used as contact electrodes are not perfectly metallic, the surface charges induced by the net polarization are not completely screened by the graphite stripes, so the depolarization electric field is not zero in the tunneling insulator. Assume that the tunneling insulator is uniformly polarized, and thus the polarization $\boldsymbol{P}$ produces a surface charge density, $\pm\sigma_P = \pm|\boldsymbol{P}|$. We first consider the 1-D screening model where the an infinite surface locates at $x = 0$ with the surface charge density of σ_P. Following the basic concept of Thomas-Fermi model, we used a simple Fermi gas model to describe the electron density in graphite:

$$\rho = 2\frac{1}{(2\pi)^3}\left(\frac{4}{3}\pi k_F^3\right) \tag{S2}$$

where k_F is the Fermi wave vector, and the Fermi energy $E_F = \frac{\hbar^2 k_F^2}{2m^*}$. m^* is the effective mass of graphite. If the temperature is extremely low, we can approximately assume the chemical potential to equate to the electric potential and then:

$$e\Delta\rho = \frac{3e\rho}{2E_F}\Delta E_F = \varepsilon_M k_0^2 \Delta\phi \tag{S3}$$

where:

$$k_0 = \sqrt{\frac{3e^2\rho}{2\varepsilon_M E_F}} = \sqrt{\frac{me^2 k_F}{\varepsilon_M \pi^2 \hbar^2}} \tag{S4}$$

where ε_M is the electric permittivity of graphite. The approximation may now be inserted into Poisson's equation in graphite:

$$\left[\nabla^2 - k_0^2\right]\phi(x) = 0 \tag{S5}$$

The solution of the above equation is the exponential decreasing potential:

$$\phi(x) = \frac{\sigma_s d}{\varepsilon_M} e^{-\frac{|x|}{\delta_0}} \tag{S6}$$

and the screening length δ_0 can be obtained by:

$$\delta_0 = \frac{1}{k_0} \tag{S7}$$

Based on the superposition principle of the electric potential, the potential profile across the junction in the real case can be described as:

$$\varphi(x) = \begin{cases} \frac{\sigma_s \delta_1}{\varepsilon} e^{-|x|/\delta_1}, \ x \leq 0 \\ \frac{\sigma_s \delta_1}{\varepsilon} - \frac{\sigma_s(\delta_1+\delta_2)}{\varepsilon d} x, \ 0 \leq x \leq d \\ -\frac{\sigma_s \delta_2}{\varepsilon} e^{-|x-d|/\delta_2}, \ x \geq 0 \end{cases} \tag{S8}$$

where d is the thickness of the tunneling barrier, δ_1 and δ_2 are the screening lengths in the left and right graphite electrodes, and σ_s is the screening charge per unit area. The screening charge σ_s can be obtained from the continuity of the electrostatic potential:

$$\sigma_s = \frac{dP}{\frac{\varepsilon_I}{\varepsilon_M}(\delta_1 + \delta_2) + d} \tag{S9}$$

where ε_I is the electric permittivity of CrOCl. In our case, the additional polarization generated from the antiferroelectric order only accounts for a small part of the whole polarization under the electric field. We here use the dielectric experiment results to describe the additional polarization, that is, ε_I = 3.06 for the unpolarized state and 3.07 for the polarized state. The band gap of CrOCl is $\sim$ 2.76 eV, obtained by our DFT calculations[1]. For graphite, we take $\varepsilon_M \sim$10, $m^* \sim 0.05 m_e$ and $v_F \sim 10^6$ m/s as the general material parameters. The calculated screening length of graphite is $\delta \sim$4.39 nm. Consequently, we can obtain the tunneling current using the integration:

$$I(V) \sim \frac{4\pi e}{\hbar} \int f_h(E) T(V, E) f_e(E - V) dE \tag{S10}$$

where $T(V, E)$ is the transmission coefficient for a single electron with the incident energy of E moving from the source graphite electrode to the $+V$ electrode. It can be calculated from a continued multiplication of the transfer matrix:

$$T(x) = \begin{pmatrix} \left(1 + \frac{q'}{q}\right) e^{(q-q')x} & \left(1 - \frac{q'}{q}\right) e^{(q+q')x} \\ \left(1 - \frac{q'}{q}\right) e^{-(q+q')x} & \left(1 + \frac{q'}{q}\right) e^{-(q-q')x} \end{pmatrix} \tag{S11}$$

where q is the wave vector of the eigenstate at the location x obtained from Schrödinger equation, and q' is that of the former step. Utilizing the above equations, we calculated the $I - V$ curves of the 12L-CrOCl tunneling junction (similar to the thickness of the devices reported in the main text) in the unpolarized antiferroelectric state and the polarized state. The tunneling current values are normalized dividing by the maximum current in the antiferroelectric state. As shown in Fig. S13b, the two curves deviate slightly from each other. Following the definition in the experimental results, we obtain the simulated current polarization ρ by:

$$\rho = \frac{I_{\mathrm{FE}} - I_{\mathrm{AFE}}}{I_{\mathrm{FE}} + I_{\mathrm{AFE}}} \tag{S12}$$

The maximum value of the simulated ρ is $\sim$ 1%, in good accordance with the experimental value reported in the manuscript. The oscillations are caused by the interference effect between the two graphite/CrOCl interfaces in the Fowler-Nordheim (F-N) tunneling regime, and they should not be measured experimentally as the real energy profile doesn't contain such sharp transitions. More intriguingly, ρ experiences a sign change at $\sim$ 4 V, which may account for the reversal of the hysteresis at $\sim$ 5.8 V in our experiments (Fig. S8,S9 and S11). At lower voltages, the tunneling current for the polarized state is larger. The ramping electric field acts as an electric excitation to tilt the electric dipoles, while the magnetoelectric effect tends to relax the system to the antiferroelectric ground state, resulting in a clockwise hysteresis. However, in the F-N tunneling regime, the tunneling current of the AFE state is larger than that of the FE polarized state, so when the sweeping electric field activates CrOCl into the polarized state, the tunneling current is lower and thus the $I - V$ curve exhibits a counterclockwise hysteresis.

Considering the fact that our calculation results quantitatively reproduce the current polarization and hysteresis behaviors in our experiments, we believe that our theoretical model captures the main physics in this system. Nevertheless, we would like to mention that the real tunneling process should be much more complicated, where many other effects, such as the interfacial states and the electrostriction effect in CrOCl, will influence the electron transmission probability. Moreover, as the magnetic order of CrOCl experiences successive phase transitions and meta-stable states, the electric order may also exhibit very complicated transitions under the external field so the $P - E$ relationship may be nonlinear. However, these effects wouldn't substantially change the results. Here we take the variation of the screening length of the graphite electrodes as an example. As shown in Fig. S13c, if we use graphite electrodes that are highly asymmetric (the density of states of the right electrode is much larger than that of the left), the energy profile of the potential barrier will be asymmetric and the current in the FE state will be much larger than the AFE state under forward bias voltages, resulting in larger ρ than the symmetric devices (Fig. S13c). This may be closer to the real situation of our measured devices.

The electric polarization in CrOCl can also play a part in the different magnetoresistance behaviors as the bias voltage varies. Under the electric field, the ↑↑↓↓ is much more polarized than the ↑↑↑↓↓ state, resulting in a sudden change of the tunneling current. The change can either be positive or negative, indicated by Fig. S13. If the graphite electrodes at both sides are symmetric, the device can exhibit symmetric magnetoresistance at positive and negative voltages, as have been observed in Fig. S14. In contrast, if the graphite electrodes are highly asymmetric, they will produce different additional potential barriers under positive and negative voltages, giving rise to asymmetric magnetoresistance behaviors (Fig. S15). However, the change of magnetoresistance is also largely influenced by the magnetic order and the magnetostriction effect upon the magnetic phase transition, so it's difficult to provide a quantitative model that perfectly coincide with our data. We believe the phenomenological theory here is the most reasonable.

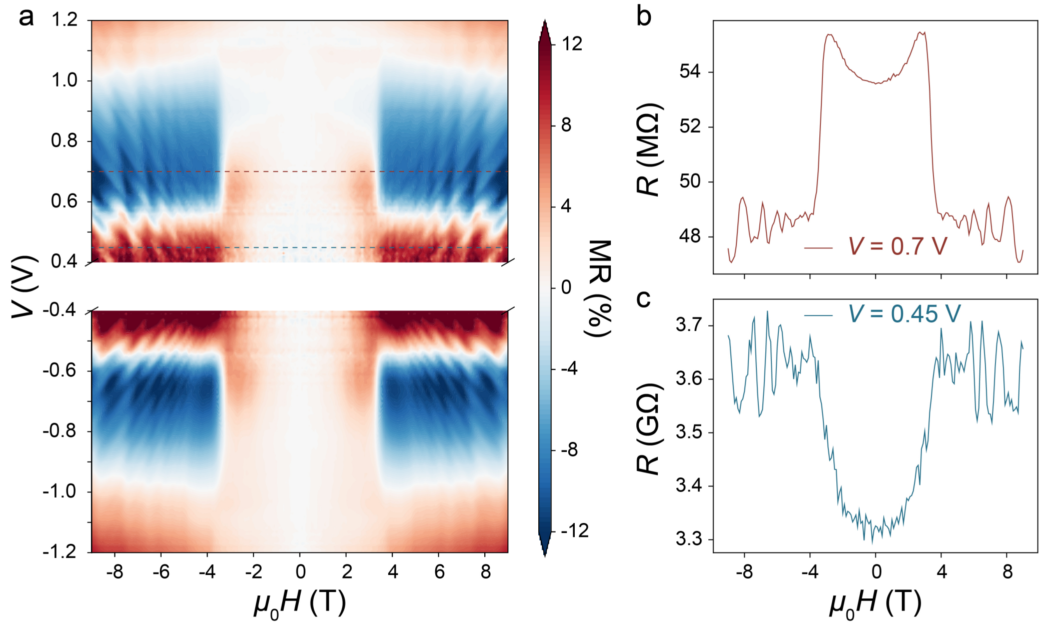

Fig. S14 Symmetric magnetoresistance (MR) behavior in device S6 (4-L CrOCl). (a), 2D map of magnetoresistance MR $= (R(B) - R(0\,\mathrm{T}))/R(0\,\mathrm{T})$ as a function of out-of-plane magnetic field and bias voltage. The horizontal dashed lines mark the bias voltage of 0.7 V and 0.45 V. **(b,c),** The resistance *versus* out-of-plane magnetic field of the CrOCl tunneling device at 0.7 V (b) and 0.45 V (c) extracted from the dashed lines in (a). As can be seen, the magnetic phase transition shows an upward magnetoresistance step at low voltages and a downward magnetoresistance step at high voltages, which are symmetric for positive and negative voltages. The varing magnetoresistance at different voltages can be resulted from the electric field induced polarization. As pointed out earlier, the electric dipoles in the ↑↑↓↓ phase can be tilted by the electric field, while the ↑↑↑↓↓ is rather immune to the external electric field. Consequently, the tunneling barrier difference between the two phases varies with the bias voltage, leading to opposite magnetoresistance behaviors at different voltages.

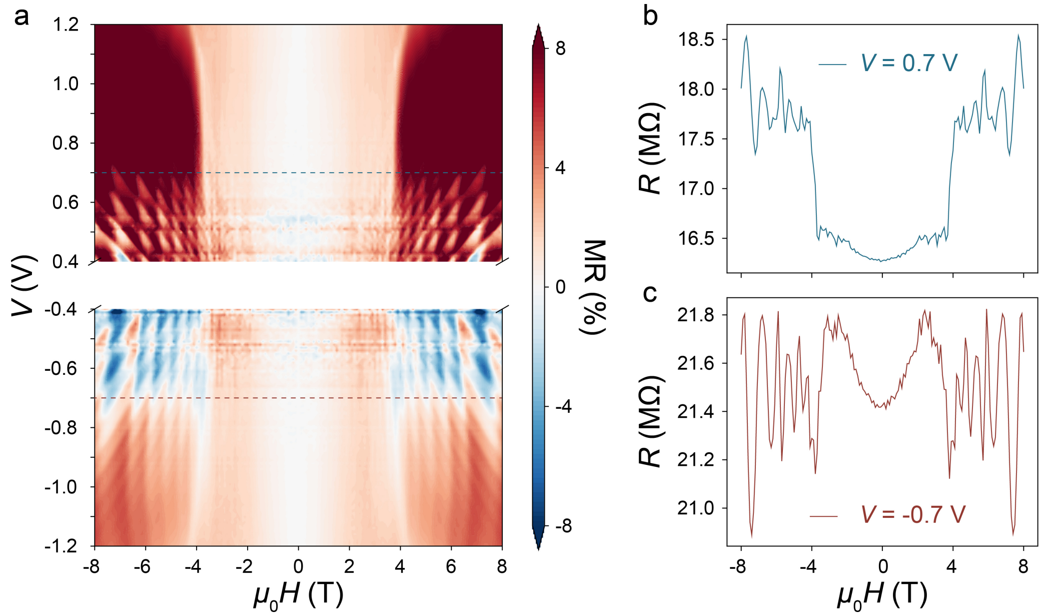

Fig. S15 Asymmetric magnetoresistance (MR) behavior in Device S2 (4-L CrOCl). (a), 2D map of MR as a function of out-of-plane magnetic field and bias voltage. **(b,c),** The resistance *versus* out-of-plane magnetic field of the CrOCl tunneling device at 0.7 V (b) and −0.7 V (c) extracted from the dashed lines in (a). The magnetic phase transition shows an upward magnetoresistance step at positive voltages and a downward magnetoresistance step at negative voltages. The asymmetric magnetoresistance is probably resulted from the asymmetry of graphite contacts. The incomplete screening in graphite electrodes gives rise to an additional electrostatic potential at the barrier/graphite interface (> 0 when P points towards the interface and < 0 when P points away from the interface). Consequently, if the graphite contacts at the two sides of CrOCl are highly asymmetrical, they will produce different additional potential barriers under positive and negative voltages, respectively. A lower additional barrier should result in positive magnetoresistance (b), while a higher additional barrier should result in negative magnetoresistance (c).

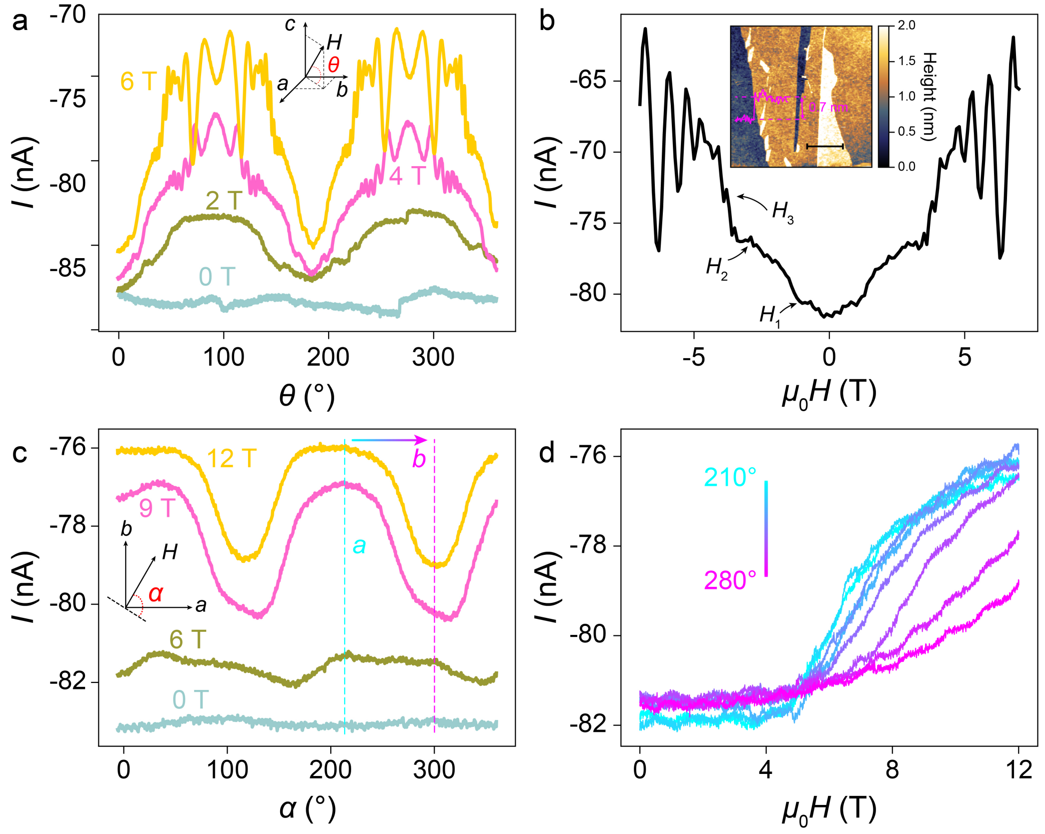

Fig. S16 Characterization of single-layer CrOCl. (a), Angle-dependent tunneling currents under different external fields. The external field is rotated along an in-plane axis to the *c*-axis. θ is the angle between the external field and the in-plane axis. **(b),** Tunneling current versus the external field along the *c*-axis. The transition fields of CrOCl are labelled by the black arrows, where H_1 and H_2 marks the beginning and the end of the spin-flop transition and H_3 marks the transition to the ↑↑↑↓↓ phase. The inset shows the atomic force microscope height image of the exfoliated single-layer CrOCl. The black scale bar is 1 μm. **(c),** The tunneling current on the dependence of the in-plane field direction at different external fields. **(d),** Tunneling current versus in-plane field as the field points towards different in-plane directions. The transition field gradually increases as the external field deviates from the a-axis. All the data were obtained in device 2 with a bias voltage of -0.018 V.

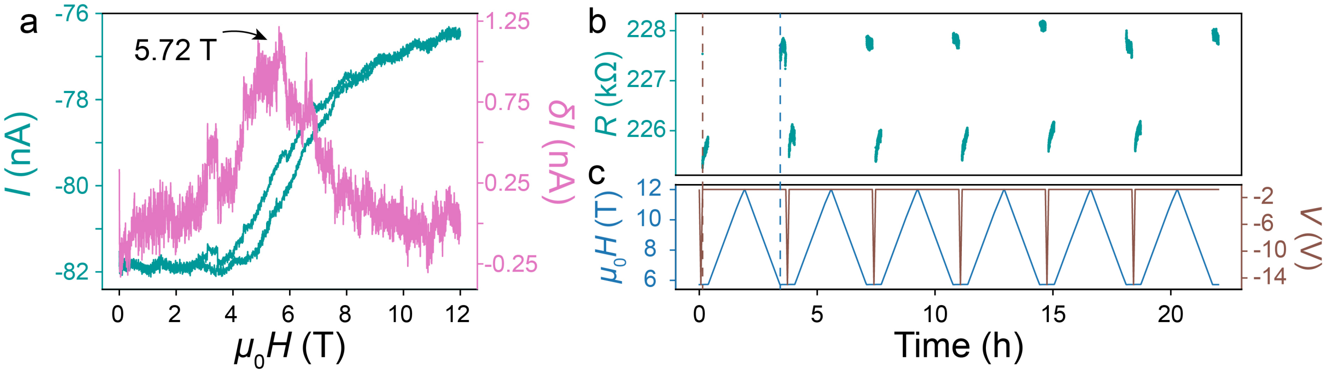

Fig. S17 Magnetoelectric coupling in a single-layer CrOCl tunneling device (device 2). (a), $I-B$ curve of the CrOCl tunneling device at 2 K with the external field along with the *a*-axis. The pink curve shows the differential current $\delta I = I(B_{\mathrm{down}}) - I(B_{\mathrm{up}})$. The critical field where δI reaches a maximum is annotated by the black arrow. **(b,c),** Tunneling current (b) after alternating electric and magnetic excitations. The corresponding magnetic and electric fields *versus* time are plotted in (c). The device was parallel-connected with a 10 MΩ protection resistor.

In order to confirm that the magnetic ground state, the electric ground state, and the magnetoelectric coupling effect of CrOCl persists down to the monolayer, we performed a series of tunneling current measurements similar to the multi-layer devices, as shown in in Fig. S16 and S17. We identified the exfoliated single-layer CrOCl by the atomic force microscopy (AFM). An AFM height image of a single-layer CrOCl flake which was used to construct device 2 is shown in the inset of Fig. S16b. The thickness of the flake is ∼0.7 nm, in accordance with the DFT calculations[8,9] of the layer distance and the previous report[10]. As shown in Fig. S16a, when the external field was rotated from an in-plane axis to the *c*-axis, the tunneling current shows a 180° periodic symmetry. The magnetic phase transitions occur at the lowest field when the external field points to the *c*-axis, evidencing that the easy axis of single-layer CrOCl is still out-of-plane. The $I-B$ curve under the out-of-plane field is shown in Fig. S16b, where the spin-flop transition and the transition to the ↑↑↑↓↓ phase can be clearly resolved. The

transition fields are labelled as H_1, H_2 and H_3, which mark the beginning, the end of the spin-flop transition and the transition to the ↑↑↑↓↓ phase, respectively. The values of the transition fields are also close to our previous results of the multi-layer tunneling devices[1].

The response of the tunneling current to the in-plane field was also measured, presented in Fig. S16c-d. When the external field is rotated in the *ab*-plane of the sample, the tunneling current shows an exactly 180° periodic symmetry (Fig. S16c). This evidences the persistence of the C_2 in-plane symmetry of single-layer CrOCl, characteristic of the one-dimensional stripy magnetic order. When the field points to the *a*-axis, the transition to the *a*-↑↑↑↓↓ phase occurs at ∼5.5 T and when the field direction rotate toward the b-axis, the transition field gradually increases and finally vanishes in our measurable field range. In summary, all the transition behaviors of the single-layer CrOCl are consistent with the reported multi-layer samples[1]. Our DFT calculations of the magnetic ground state and the resulted atomic distortions are also performed both in bulk and single-layer CrOCl and obtained the same results (Fi.g S1). Consequently, we conclude that the magnetic order, as well as the spin-induced electric transition, maintains the same for single-layer CrOCl.

The manipulation of the electric states in single-layer CrOCl is presented in Fig. S17. Similar to the multi-layer device reported in the manuscript, we obtained the maximum hysteresis at B_0 = 5.72 T by subtracting the current of the *B*-up curve from that of the *B*-down curve. Likewise, by alternately applying the magnetic and electric excitations, we can repeatedly realize the manipulation of the resistance state between two values, 225.5 kΩ and 227.5 kΩ. As a result, the same operation principles, as well as the microscopic mechanisms, apply to single-layer CrOCl. The only difference is that in the single-layer device, the difference between the highest and lowest resistance states is lower, so it is difficult to realize multi-level resistance in a single device. This is probably caused by two reasons. Firstly, the tunneling barrier of the single-layer CrOCl is much thinner, naturally resulting in lower magnetoresistance. Secondly, the electric dipoles in a monolayer can only host in-plane interactions with each other in the absence of neighboring layers, which may produce less metastable states during the phase transition. Nevertheless, the underlying physical picture remains the same.

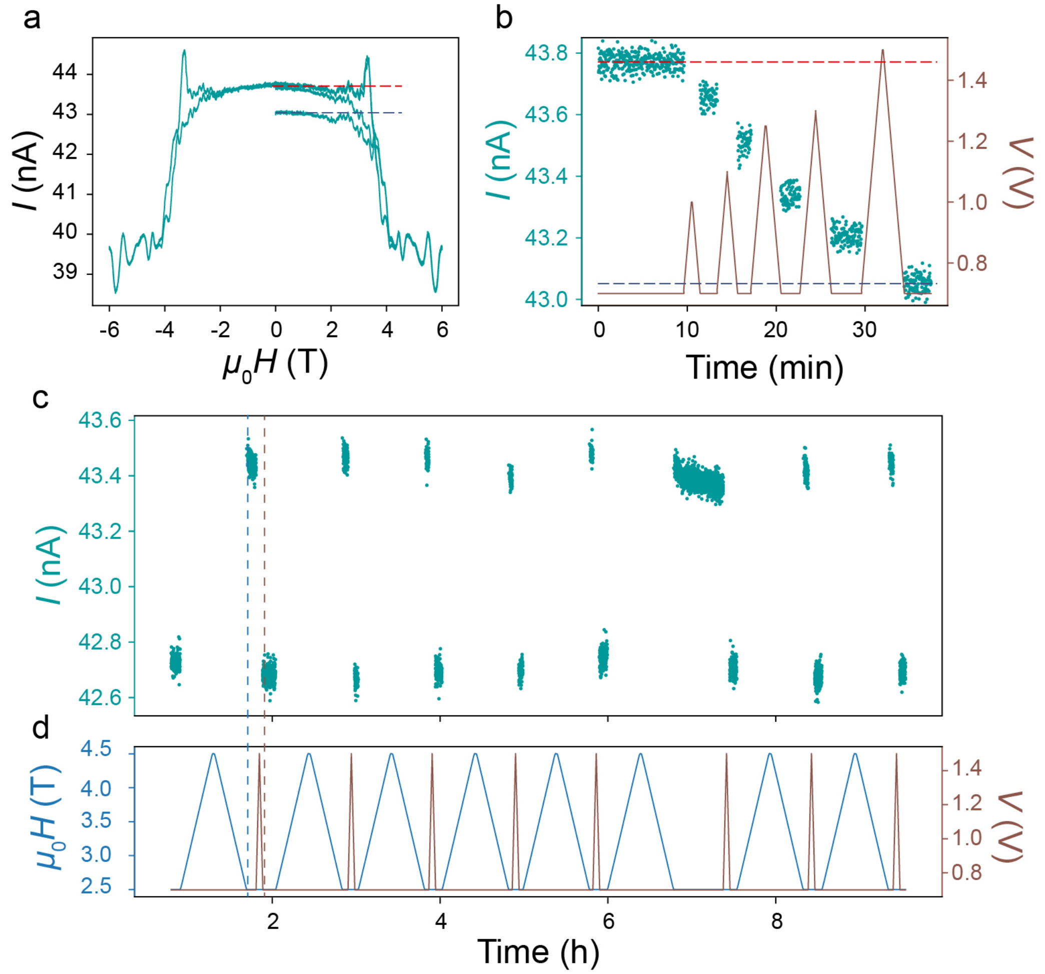

Fig. S18 Magnetoelectric coupling at zero magnetic field in device S7. (a), $I - B$ curve of the CrOCl tunneling device at 2 K. After sweeping back to zero field from a large out-of-plane magnetic field (larger than B_s), the device reaches a high-current excited state (red dashed line) at zero field. The new state should be another metastable state in the ↑↑↓↓ phase, which is achieved after a series of structural transitions accompanied with the magnetic transitions. **(b),** Tunneling current over time after each electric excitation at zero magnetic field. The red and blue dashed lines represent the current values of the excited state and the ground state at 0 T extracted from (a), respectively. **(c,d),** Tunneling current (c) after alternating electric and magnetic excitations. The corresponding magnetic and electric field *versus* time are plotted in (d). The initial field is set to 2.5 T for a lower magnetic excitation, which does not affect the current states substantially.

References

[1] Gu, P. *et al.* Magnetic phase transitions and magnetoelastic coupling in a two-dimensional stripy antiferromagnet. *Nano Lett.* **22**, 1233–1241 (2022).

[2] Song, T. *et al.* Giant tunneling magnetoresistance in spin-filter van der waals heterostructures. *Science* **360**, 1214–1218 (2018).

[3] Jang, S. W. *et al.* Hund's physics and the magnetic ground state of CrOX (X= Cl,Br). *Phys. Rev. Mater.* **5**, 034409 (2021).

[4] Kohlstedt, H., Pertsev, N., Contreras, J. R. & Waser, R. Theoretical current-voltage characteristics of ferroelectric tunnel junctions. *Phys. Rev. B* **72**, 125341 (2005).

[5] Tsymbal, E. Y. & Kohlstedt, H. Tunneling across a ferroelectric. *Science* **313**, 181–183 (2006).

[6] Zhuravlev, M. Y., Sabirianov, R. F., Jaswal, S. & Tsymbal, E. Y. Giant electroresistance in ferroelectric tunnel junctions. *Phys. Rev. Lett.* **94**, 246802 (2005).

[7] Chanthbouala, A. *et al.* A ferroelectric memristor. *Nat. Mater.* **11**, 860–864 (2012).

[8] Miao, N., Xu, B., Zhu, L., Zhou, J. & Sun, Z. 2d intrinsic ferromagnets from van der waals antiferromagnets. *J. Am. Chem. Soc.* **140**, 2417–2420 (2018).

[9] Zhang, F. *et al.* Super-exchange theory for polyvalent anion magnets. *New J. Phys.* **21**, 053033 (2019).

[10] Zhang, T. *et al.* Magnetism and optical anisotropy in van der waals antiferromagnetic insulator crocl. *ACS nano* **13**, 11353–11362 (2019).
