# Supplementary material for: Multi-state data storage in a two-dimensional stripy antiferromagnet implemented by magnetoelectric effect
Source: Nat Commun. 2023 Jun 3;14:3221. doi: 10.1038/s41467-023-39004-4 (PMC10239514; doi:10.1038/s41467-023-39004-4)
Supplement: Supplementary file 3 — Source Data [file 41467_2023_39004_MOESM3_ESM.zip › Source Data/Source Data Guide.docx]

The Source Data ZIP file includes three source data files (**Figure 1.xlsx, Figure 2.xlsx,** and **Figure 3.xlsx**).

- Legend for **Figure 1.xlsx** file**:**

This file includes source data for Figure 1 in the main text.

- Legend for **Figure 2.xlsx** file**:**

This file includes source data for Figure 2 in the main text.

- Legend for **Figure 3.xlsx** file**:**

This file includes source data for Figure 3 in the main text.
